# Two replications of an investigation on empathy and utilitarian judgement across socioeconomic status

**DOI:** 10.1038/sdata.2016.129

**Published:** 2017-01-17

**Authors:** Sarah Babcock, Yixian Li, Vanessa M. Sinclair, Clint Thomson, Lorne Campbell

**Affiliations:** 1 Western University, Department of Psychology, London, Ontario, Canada N6G 2V4

**Keywords:** Human behaviour, Research data

## Abstract

Research by Côté, Piff, and Willer (2013) found that through the induction of empathy in an experimental condition, the association between socioeconomic status (SES) and utilitarian moral judgment was diminished. Participant self-reported income interacted with experimental condition such that high SES participants who empathized with a disadvantaged group member redistributed fewer experimental dollars during an online task at the losing member’s expense. This suggests that lower levels of empathy could help explain utilitarian decision-making in high SES individuals. Two pre-registered, high-powered replications were conducted in order to assess the magnitude and reliability of this finding. While the first replication attempt failed to uncover the effect, the second attempt found a pattern consistent with the original study. A meta-analysis of the replication attempts with the original author's interaction effects was conducted. The confidence interval of the meta-analytic effect suggests that the true effect size may be as robust as reported by the original authors, or may be close to zero. Implications of the results found in the replication attempts are discussed.

## Background and Summary

Socioeconomic status (SES), which stratifies individuals based primarily on their access to resources, has long been thought to correspond to differences in reasoning. However, the moral decision-making of individuals who belong to different social classes has only recently come under scientific scrutiny. Research has suggested a number of negative effects are associated with high SES, which include acting less generously^1^ and demonstrating overt unethical behaviour, such as cutting off other cars or pedestrians while driving, as well as lying in negotiations^2^.

A recent study by Côté, Piff, and Willer^3^ found that those of high social class tend to use utilitarian judgment, a moral decision-making strategy based on choosing the action that produces the greatest benefit for the most individuals. Although utilitarian decisions maximize benefits for the majority of people, they come at a cost to an unlucky few. Interestingly, in a follow-up to the study that produced this initial finding, the authors reported that the propensity of high-status individuals to take resources from one to help many is mitigated when they are made to empathize with the disadvantaged. The authors therefore suggest that a lack of empathy for those on the losing end may be a contributing factor to this moral decision-making strategy^3^.

To our knowledge, there had been no experimental evidence on the interrelation of SES, utilitarianism, and empathy prior to this work^3^. As decisions rooted in utilitarian judgment benefit the many at a cost to the few, the reported effects have potentially far-reaching sociopolitical implications, particularly for groups that stand to be disadvantaged. A discussion on the righteousness of a utilitarian moral framework is beyond the scope of this paper, but it will suffice to say that the means through which high SES individuals make moral decisions is a point worth investigating. As the recent work of Côté *et al.*^3^ suggests a possible mechanism—the induction of empathy—that may affect the moral decision-making process, it is not surprising that these findings have been referenced by works within the fields of criminology^4^, industrial-organizational psychology^5^, and social psychology^6^, among others.

At the time of writing, Côté *et al.*’s^3^ work has been cited a total of 43 times according to Google Scholar, and 21 according to PsycINFO. However, the finding that the induction of empathy diminishes utilitarian decision-making in high SES individuals has yet to be replicated, and the authors importantly note that the effect size of their result was small: SES, the empathy-inducing manipulation, and their interaction jointly explained only 6% of the total variance in participants’ moral decision-making^3^. In order to determine the reliability and magnitude of the reported effect—which are primary goals of scientific investigation—direct replications of studies are required. The replication process contributes to the creation of scientific consensus and enhances understanding of phenomena; this is especially important for work with potential societal implications such as these. Therefore, in the present research, we have conducted two pre-registered direct replications of Côté *et al.*’s study with larger sample sizes and greater statistical power to test the original authors’ hypothesis. The hypothesis was that empathy accounts in part for the association between social class and utilitarian judgement; specifically, in a control condition, there would be a positive association between social class and utilitarian judgment, while this association would be reduced or eliminated in a condition wherein empathy is elicited.

## Methods

Our replication study was designed to use the same measures, procedure, and sampling population as the original. We contacted the first author of the original study—Dr Stéphane Côté—directly prior to starting the process to discuss the methodological approach and sampling requirements. In this correspondence, we provided a copy of our replication plan and requested his review and feedback to ensure we were correctly aligning our study with the original. Based on his responses and collaboration, we were able to obtain access to the same measures that were used in the initial project. We therefore used the exact instructions and wording for all measures, as well as the information provided to us regarding the order and layout of the instructions. Two additional questions were added to our measures that deviated from the original study materials. The first question was added to the end of our questionnaire (in both replication attempts) to assess whether participants suspected the hypothesis of the study, a factor that could influence the results. The second question was added only in the second replication attempt, and asked participants if they had completed this study (or a similar study) as part of a previous MTurk assignment. Lastly, we increased the payment participants received in comparison to the original study (from $0.50 to $1.00). We made no other deviations from the original materials. In total, we conducted two independent replication attempts; the first took place in November 2015, and the second in February 2016. Prior to beginning data collection, the replication attempts were preregistered on the Open Science Framework (OSF), and all correspondence, materials, and analytic plans were published on the study page: http://www.osf.io/sgjpy.

### Code availability

In addition to the analytic plan and study materials, after the completion of data collection and analyses, both the data file and syntax files were placed on the OSF. All components are fully available without restrictions, and are accessible on the OSF page of this study as provided above. The analyses were predominately conducted using the statistical software IBM SPSS Statistics (23); the data and syntax files are in the *.sav* and *.sps* file formats. The exception to this was the meta-analysis of the interaction effect using unstandardized regression coefficients, which was conducted using the metaphor package in *R*^7^. Information regarding use of the study code, data file, and syntax can be found in the Data Records section below.

### Participants

The original study sample size was 91. For our first replication, the target sample size was 230, calculated by multiplying the original sample size by 2.5. This increase in sample size was to ensure high statistical power and was based on evidence presented by Simonsohn^8^ on optimal sample size for replication studies. In an effort to conduct a fair test of the hypothesis, we also collected a second sample for an additional replication attempt. The intention of this replication project was to examine whether the pattern of findings of the original paper hold in independent samples. Therefore, after our first replication attempt failed to replicate the findings, in the interest of objectivity we decided to test the hypothesis again in an additional sample to give the original study findings a fair chance at replication, and to confirm that our first sample findings were not due to random chance. For this second attempt the target sample size was 300; this increased sample size was set in an attempt to further increase study power. Participants were collected using the same recruitment method—online, via Amazon’s Mechanical Turk (MTurk)—and sampling framework (US citizens, 18 years and older) as the original study.

#### Replication study #1

A total number of 257 people participated in our study. Twenty-four participants were excluded from the sample because they were not residents of the United States; six were excluded for choosing the ‘Prefer not to say’ option, and one for providing no choice, on the household income question (which was an independent variable). Of the remaining 226 participants (129 men, 96 women, and one who preferred not to disclose), ages ranged from 18 to 70 (*M*=35.36, s.d.=11.25). Overall, 172 (76%) participants were Caucasian, 21 (9%) were Asian American, 19 (8%) were African American, 11 (5%) were Hispanic, and three (1%) selected another category. The median household annual income value (measure of social class) was 4 (s.d.*=*1.80) corresponding to the $35, 001–$50, 000 range. Religiosity had a mean value of 3.14 (s.d.*=*2.14) on a scale from 1 (not at all religious) to 7 (very religious) and political orientation had a mean value of 3.53 (s.d.=1.78) on a scale from 1 (very liberal) to 7 (very conservative).

#### Replication study #2

A total number of 301 individuals participated in our study. Six participants were excluded because they were not residents of the United States. Of the remaining 295 participants (128 men, 167 women) ages ranged from 18 to 78 (*M*=34.85, s.d.=11.15). In total, 216 (73%) participants were Caucasian, 35 (12%) were Asian American, 17 (6%) were African American, 16 (5%) were Hispanic, and three (4%) selected another category. The median value of household annual income was 4 (s.d.=2.02). Religiosity had a mean value of 3.36 (s.d.=2.06) and political orientation had a mean value of 3.51 (s.d.=1.61).

The demographic characteristics of both replication samples were similar to that of the original study, though the distribution of gender in sample 1 contained a proportionally higher amount of men than either the original study or sample 2. In the Technical Validation section below, demographic information for the original study, alongside the replication attempts, is provided for comparative purposes. An important note regarding the exclusion of participants is that a significantly higher proportion of cases were excluded in sample 1 (9.5%) in comparison to sample 2 (1.9%). In our first replication study, we did not set strict limitations on the Qualtrics platform to allow only for US IP addresses; we therefore had a number of participants enrolled in the study who were not US residents whom we had to exclude. We rectified this setting in our second replication attempt. We believe that this modification accounted for the significant reduction in the number of excluded cases in the second sample.

### Materials

#### Demographic questionnaire

Administered to collect information on the control variables (age, gender, ethnicity, political orientation, and religiosity), as well as household income, which was used as a measure of social class.

#### Allocation task

This task described a scenario and informed participants that at a later stage in the experiment, they would have the choice of taking away up to 5 experimental dollars from one ‘lose’ member of their group in order to allocate an increased amount of experimental dollars to three ‘keep’ members (not including themselves). The amount taken was used a measure of utilitarian judgment. The exact wording of the instruction, used in the original study and the current replication attempts, is as follows:

'You have been paired with 4 other participants to create a group of 5 members. Your decisions in this part will be anonymous, and at no point during or after the experiment will anyone learn the identity of anyone they were paired with. This exercise will involve 'experiment dollars'; each experiment dollar is worth 1 entry in a raffle to give away a $50 prize to one of the participants in the study. In each group of 5, members play one of the following roles. The DECIDER makes decisions that will impact the experiment dollars earned by the other members. The LOSE member will potentially lose experiment dollars to be converted into chances of winning a $50 prize. The other three members will be KEEP members -- these members will potentially win experiment dollars to be converted into chances of winning a $50 prize.

From random assignment performed by the survey when you clicked on the link, you have been selected to be the decider in today’s exercise. Your task is to make the following decision that will impact the other members of your group: All members have received 5 experiment dollars. However, you can decide to reduce the number of experiment dollars given to the lose member of your group in order to benefit the keep members. Specifically, for each 1 experiment dollar that you take away from the lose member, 2 experiment dollars will be added to each of the keep members’ payment.

For example, if you take away 1 experiment dollar from the lose member, each keep member will gain 2 experiment dollars. Thus, at the end, the lose member would earn 4 experiment dollars (5 experiment dollars initially minus the 1 that they will lose), and each keep member would earn 7 experiment dollars (5 experiment dollars initially plus the 2 that they will gain). If you take away 2 experiment dollars from the lose member, each keep member will gain 4 experiment dollars. Thus, the lose member would earn 3 experiment dollars (5 experiment dollars initially minus the 2 that they will lose), and each keep member would earn 9 experiment dollars (5 experiment dollars initially plus the 4 that they will gain). And so on...'

#### Empathy induction instruction

Participants in the empathy condition were asked to 'concentrate on trying to imagine how the ‘lose’ member feels and how your decision will influence him or her’, and to write at least three sentences describing the feelings and wellbeing of the ‘lose’ member.

#### Empathy induction check

To verify that empathy was manipulated as intended, we administered a three-item scale. Participants indicated how much they felt compassionate, moved, and sympathetic towards the ‘lose’ member on a scale from 1 (not at all) to 7 (very much).

All study materials used are available in the Replication Protocol document registered on the OSF study page.

### Procedure

The two replication attempts were identical in study design and materials. The exceptions to this were the following: (1) the target sample sizes were increased, and (2) in the second attempt, the survey was modified to include a question asking participants if they had ever completed this study or similar study in the past, in order to prevent ‘ballot box stuffing.’

The study was completed via the Qualtrics online survey platform, which participants can access remotely from their personal computer or mobile device. Once enrolled in the study, all participants completed a set of demographic questions. This was imperative to ensure that all sample criteria were met and to measure the independent variable of income. All participants then saw the same allocation task instructions. After reading the instructions, participants were randomly assigned to either the empathy or control condition. In the first sample, 112 participants were assigned to the empathy condition and 114 to the control. In the second sample, 157 participants were assigned to the empathy condition and 138 to the control. Those in the empathy condition were presented with the empathy-inducing instructions, asking them to describe their feelings about the ‘lose’ member. Participants in the control condition did not complete this task.

All participants were then asked to complete a measure of empathy as a manipulation check. Following this, all participants completed the allocation task, in which they were asked to indicate how many experimental dollars they would take away from the ‘lose’ member. One additional component of our procedure (that differed from the original) was the decision to ask participants whether they suspected the study hypothesis, and to what degree this influenced their choices. This was done after all original study components were completed.

Finally, participants were thanked and debriefed. All participants were then compensated for their participation in the study. Based on feedback from the original author and with consideration to the number of years that have passed between the original study and these replications, the compensation for our study was increased to $1.00 (as compared to $0.50 in the original study).

## Data Records

The data for the replication samples are available on the OSF project page, outlined in Table 1 below. This OSF components page contains: the complete dataset (including data from both replication samples), the complete output files (in .spv and .pdf format), the complete syntax file (.sps format), and one *R* code file for meta-analysis. The dataset file contains the data from both samples. The sample code was provided in the dataset and descriptions for the variables in the dataset can be seen in the ‘Value’ column for each variable in ‘Variable View’. Instructions regarding the use of the syntax for separate samples (replication 1 or replication 2), or the combined sample, can be found within the syntax file (Data Citation 1).

It is worth noting that data from the excluded participants in replication sample 1 were deleted from the original data file as a part of the initial data cleaning process, and were unfortunately unable to be recovered (and therefore included) in the data file provided on the OSF components page. However, we recognize that deleting excluded participant data from the original data file was not an ideal practice in terms of research transparency and future replication endeavours. We therefore revised the approach for the second replication attempt, ensuring to include data from all participants in the data set, and specifying the excluded participants using a data filter instead (see the syntax for details).

## Technical Validation

To ensure reliable and unbiased assignment, participants were randomly assigned to one of two experimental conditions via the randomization option in Qualtrics software, and we selected the option to distribute participants equally between experimental groups. However, an important note is that in the second replication sample, there is a somewhat higher proportion of cases (53%) in the Empathy condition despite our efforts to have equal participants in each experimental group. This slightly larger sample size in the empathy group is related to (1) removal of participants who initiated but failed to complete the study, and (2) the exclusion of participants based on location (i.e. non-US residents), given that there were more participants in the control condition who had to be removed based on this criteria. In addition, participants were invited to participate without any major exclusionary criteria (required only to reside in the U.S. and be 18 years or older, which was the exclusion criterion used in the original study)^3^. Our collected samples reflect both male and female adults ranging in age from 18–78. The samples closely align with the original study demographics; Table 2 below presents the demographic characteristics of all the samples for comparative purposes.

Furthermore, as a part of our replication studies, we included a measure of suspicion to assess whether participants could correctly predict what the study was attempting to investigate. This analysis was conducted for both replication samples one and two. While 10.8% of participants accurately predicted the hypothesis, filtering out these suspicious participants did not significantly change the results in either of the replication attempts, therefore these participants were not removed in the final analyses. The suspicious cases were coded by an author of the manuscript, and are included in the dataset as 1=suspicious and 0=not suspicious. The associated syntax file provides instruction on how to apply this filter.

Finally, with regards to the additional question added in replication study 2—which asked participants if they had completed this study or a similar study in the past year—the decision was made not to eliminate participants based on a response of *Yes* (which was our original intent). After reviewing the text responses (available in the dataset) that accompanied some of the *Yes* and *Not Sure* responses, it became evident that the question was phrased too vaguely to accurately determine whether or not the participant had actually completed this or a similar study, or rather had just completed other MTurk studies. The nature of the wording did not, in our opinion (and based on review of the text responses) adequately parse out those who had actually completed very similar studies compared with those who had not. For example, participants made statements such as: ‘*I did complete a money distribution survey but it was not this one. It was somewhat similar though*’, and ‘*There was a similar task to divide money but only for personal benefit*’. Therefore, we did not feel that this was an appropriate measure by which to determine the intended value of the question and opted not to eliminate any participants.

Of primary interest to us as replicators was the interaction of experimental condition (empathy versus control) and SES. In the original study, Côté *et al.* used multiple regression analyses to test the interaction between condition (a two-level categorical variable) and SES (a quasi-continuous variable)^3^. They hypothesized that in the control condition, there would be a significant positive association between utilitarian judgment and SES, and in the empathy condition, there would be no significant association between utilitarian judgment and SES.

To test the hypotheses of the original study, we used the same multiple regression analytic approach used in the original study. Identical analyses, described as follows, were conducted on the two replication samples. Dummy coding was applied to some variables as was done by Côté *et al.* before the multiple regressions were carried out^3^. Male was coded as 0 and female as 1 for gender; Caucasian was coded as 1 and other ethnic groups as 0 for ethnicity; and the control group was coded as 0 and empathy-induction group as 1 for the experimental conditions.

In Model 1, we entered the interaction term of SES and condition (computed by multiplying each participant’s value on these two variables) into a regression equation along with the main effect terms of SES and condition. The significance of the interaction term would suggest whether the regression of utilitarian judgment on SES was identical between the two conditions. Following this first regression, we then conducted simple regressions of utilitarian judgment on SES with each of the two experimental conditions. We also ran a second multiple regression model, Model 2, with demographic variables including gender, age, ethnicity, religiosity, and political orientation entered as predictors, in addition to the interaction of empathy and condition, and their main effect terms. The primary interest for our replication attempt was the effect of the interaction, therefore we took the same approach as the original authors. However, we chose not to interpret the main effects in the presence of the interaction because dummy coding was used in the multiple regression analysis^9^.

### Manipulation check

In the original study, participants in the empathy condition reported greater empathy for the ‘lose’ member of the group (*M*=4.59, s.d.=1.33) than did those in the control condition (*M*=3.99, s.d.=1.47), *t*(89)=2.03, *P*<0.05. In our first replication, participants in the empathy condition also reported greater empathy for the ‘lose’ member (*M*=5.35, s.d.=1.27) than did participants in the control condition (*M*=4.85, s.d.=1.56), *t*(223)=2.67, *P*<0.01. This effect was also observed in our second replication (*M*Empathy=5.18, s.d. Empathy=1.37; *M*Control=4.59, s.d. Control=1.64), *t*(293)=−3.33, *P*=0.001.

### Regression analyses

In the original study, Côté *et al.* reported that the interaction term of income and empathy contributed significantly to the prediction of utilitarian judgment in both Model 1, *b*=−0.34, *t*=−1.92, *P*<0.10, and in Model 2, *b*=−0.35, *t*=−2.02, *P*<0.05 (ref. 3). In our first replication, the data did not yield this result in Model 1, *b*=−0.02, *t*(222)=−0.11, *P*=0.91 or Model 2, *b*=−0.01, *t*(214)=−0.07, *P*=0.95. Please note that three participants from the first replication attempt were not included in the regression analysis for Model 2, due to missing data of their age (*N*=2) and gender (*N*=1; indicated ‘preferred not to disclose’ for the gender question). In our second replication, we found that the interaction of income and condition contributed significantly to utilitarian judgment in Model 1, *b*=−0.24, *t*(291)=−2.40, *P*<0.05, and in Model 2, *b*=−0.23, *t*(286)=−2.38, *P*<0.05. When we combined the data from our two replications, we obtained a regression coefficient for the interaction term in Model 1 that is comparable to what was found by Côté *et al.*, *b*=−0.14, *t*(517)=1.70, *P*<0.10, but not in Model 2, *b*=−0.12, *t*(509)=−1.52, *P*=0.13. Thus, while we did not obtain the same results as the original authors in our first replication, the pattern of results was consistent with the original study in the second replication, as well as in Model 1 when combining replications 1 and 2. All of the regression coefficients for Models 1 and 2 in both the original study and our replications are reported in Tables 3 and 4.

As a follow-up to obtaining a significant interaction effect in the original study, Côté *et al.* conducted simple slopes analyses examining the regression of utilitarian judgment on income in both the empathy and control conditions^3^. To adhere to the same analytical approach as the original authors, we also conducted simple slopes analyses of the regression of utilitarian judgment on income in replication 1, replication 2, and the combination of replication studies 1 and 2.

**Figure 1:**
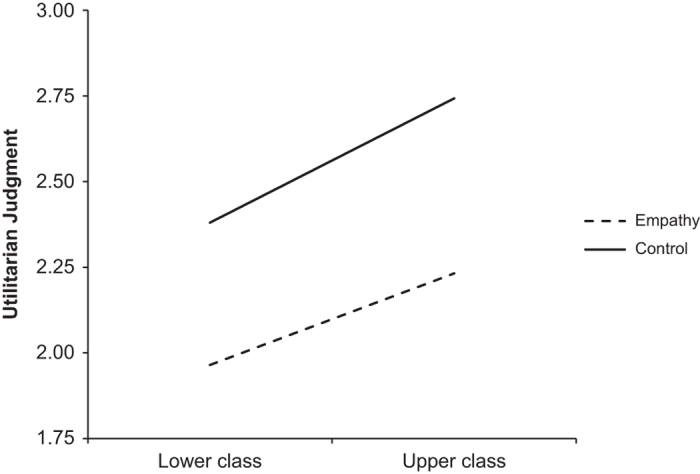
Utilitarian Judgement by Condition, Replication 1. This figure illustrates the results from our first replication attempt, showing the regressions of utilitarian judgment on social class in the empathy and control conditions. The total sample included *N*=226 (112 in Empathy Condition, 114 in Control Condition). This sample was analyzed independently of other study samples.

**Figure 2:**
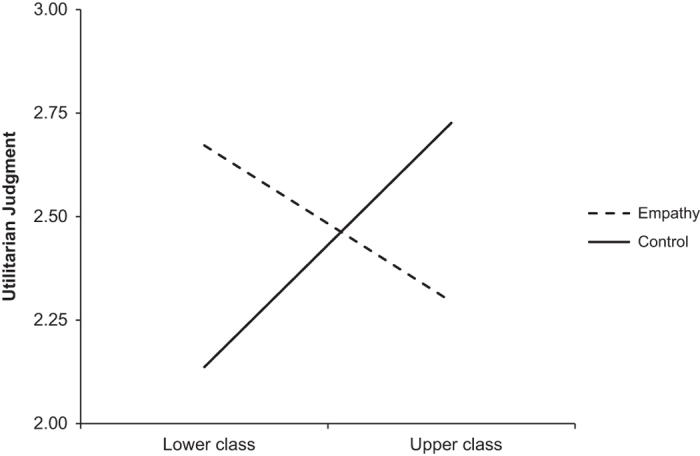
Utilitarian Judgement by Condition, Replication 2. This figure illustrates the results from our second replication attempt, showing the regressions of utilitarian judgment on social class in the empathy and control conditions. The total sample included *N*=295 (157 in Empathy Condition, 138 in Control Condition). This sample was analyzed independently of other study samples.

**Figure 3:**
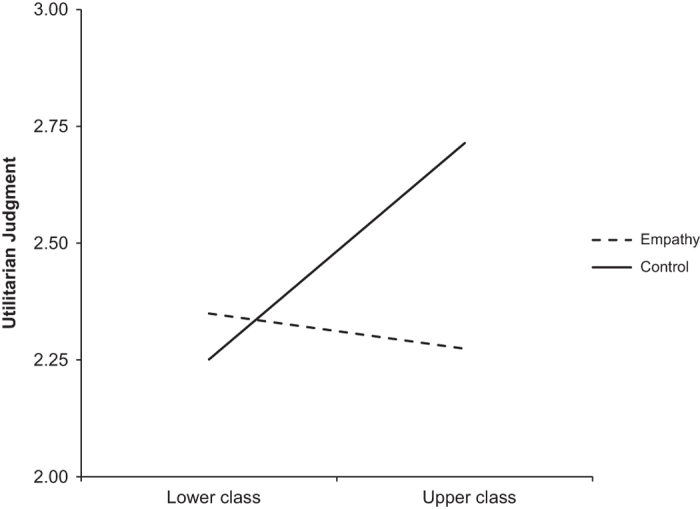
Utilitarian Judgement by Condition—Replication 1 and 2 Combined. This figure illustrates the results from our combined samples from replication attempts 1 and 2, showing the regressions of utilitarian judgment on social class in the empathy and control conditions. The total sample included *N*=521 (269 in Empathy Condition, 252 in Control Condition).

The authors of the original study reported that the regression of income on utilitarian judgment in the control condition was significant, *b*=0.27, *P*<0.05, while it was not in the empathy condition *b*=−0.06, *P*=0.63. T-values for these regression coefficients were not reported. In our first replication, we found that the regression of utilitarian judgment on income was not significant in the empathy condition, *b*=0.08, *t*(110)=0.83, *P*=0.41, or the control condition, *b*=0.09, *t*(112)=0.94, *P*=0.35. In our second replication, we found a significant regression of utilitarian judgment on income in the control condition, *b*=0.15, *t*(155)=2.16, *P*<0.05, but not in the empathy condition, *b*=−0.09, *t*(136)=−1.27, *P*=0.21. When combining the data for our first and second replications, we found a significant regression of utilitarian judgment on income in the control condition, *b*=0.12, *t*(269)=2.08, *P*<0.05, but not in the empathy condition *b*=−0.02, *t(*248)=−0.34, *P*=0.73. Thus, the interaction effects we obtained in the second replication and when combining the data from the first and second replications mirrored the results obtained by Côté *et al.*^3^, while the results in our first replication did not. The interaction effects in our first and second replications and the combination of replication 1 and 2 are represented pictorially in Figs 1,2,3.

**Figure 4:**
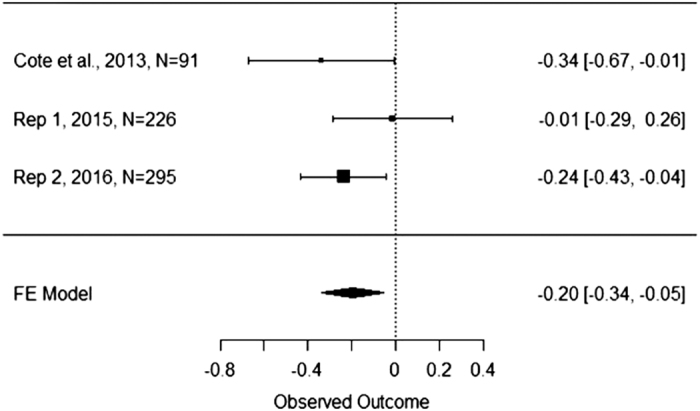
Meta−Analysis of Interaction Coefficient Across Three Studies. This figure illustrates the unstandardized coefficient and 95% Confidence Interval for the SES by Condition interaction found in the original study by Côté *et al.*^3^, replication attempt one, replication attempt two, and the meta-analytic effect.

**Figure 5:**
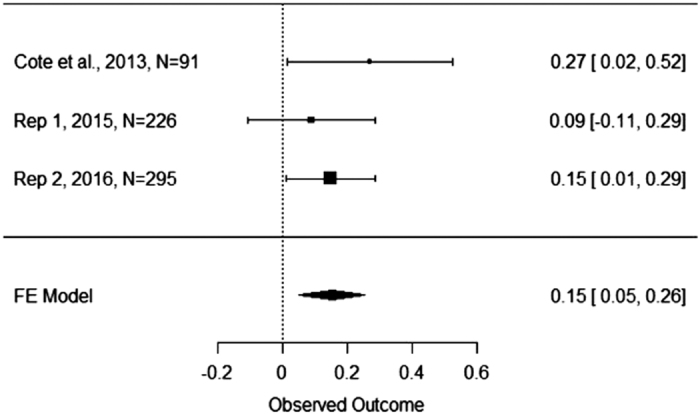
Meta−Analysis of Simple Slopes in the Control Condition. This figure illustrates the unstandardized coefficient and 95% Confidence Interval for regression of utilitarian judgment on social class in the control condition as found in the original study by Côté *et al.*^3^, replication attempt one, replication attempt two, and the meta-analytic effect.

**Figure 6:**
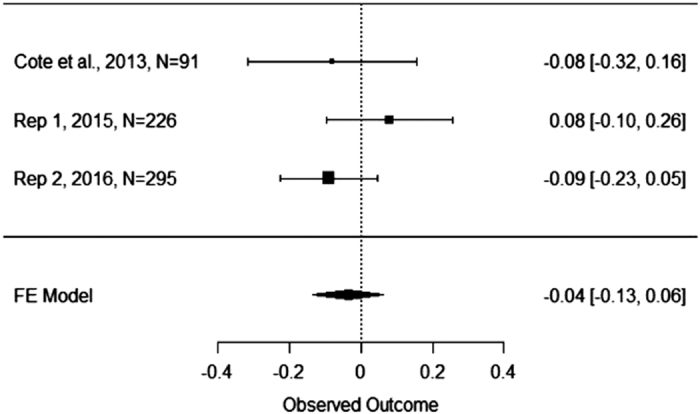
Meta−Analysis of Simple Slopes in the Empathy Condition. This figure illustrates the unstandardized coefficient and 95% Confidence Interval for regression of utilitarian judgment on social class in the empathy condition as found in the original study by Côté *et al.*^3^, replication attempt one, replication attempt two, and the meta-analytic effect.

In order to generate the most precise estimate of the interaction effect of empathy and social class on utilitarian moral judgment, we conducted a fixed effects meta-analysis on the unstandardized regression coefficients obtained both by us and Côté *et al.*^3^ Figure 4 presents the coefficients for the interaction term and their 95% confidence interval for the original study, two replication attempts, and their meta-analytic effects. The weighted mean unstandardized coefficient we obtained was −0.20, 95% CI [−0.34, −0.05]. This confidence interval suggests that the true effect could range from being near what the original authors found, or near zero. Figure 5 presents the results of a similar fixed effects meta-analysis on the unstandardized regression coefficient representing the simple slope between social class and utilitarian moral judgment in the control condition. The weighted mean unstandardized coefficient was.15, 95% CI [0.05, 0.26]. Lastly, Figure 6 presents the results of a fixed effects meta-analysis on the unstandardized regression coefficient representing the simple slope between social class and utilitarian moral judgment in the empathy condition. The weighted mean unstandardized coefficient was −0.035, 95% CI [−0.13, 0.06]. The results of the meta-analyses suggest that the interaction coefficient varied in size from moderate to small, that the coefficient was near zero in the empathy condition, and that the coefficient was reliably positive in the control condition.

## Usage Notes

In the present research, we attempted to replicate Côté *et al.*’s finding that the association between SES and utilitarian judgment can be reduced following the elicitation of empathy for a disadvantaged group member^3^. In our first replication study, we did not find support for this hypothesis. However, the original finding was supported in our second replication study and when meta-analyzing the results of both replications in combination with the original findings. While the results are inconclusive, we believe our replication studies and meta-analysis will contribute to a more complete understanding of the association between SES and utilitarian judgment.

When considering the replication samples separately, support for the phenomenon is mixed, as the results of the first replication are not consistent with the hypothesis that the association between SES and utilitarian judgment is attenuated following the induction of empathy. Thus, the failure to replicate the results of Côté *et al.*^3^ in the first sample is worth discussing. One potential explanation for the failure to replicate is sampling error. In some instances, researchers can draw a single sample with which they will obtain an inaccurate estimate of a population effect. If researchers collect more than one sample, however, they are more likely to generate an accurate estimate. It is therefore worth noting that conducting the second replication study altered our estimation of the population effect. The results of the second replication and meta-analysis suggest that the effect found in the original study could exist, although it may not be as large as originally reported. We also found that the gender distribution of sample 1 differed from that of the original study and sample 2, with sample 1 having a larger proportion of men (57%, compared to 38 and 43%, respectively). This gender difference may have played a role in the difference in results between samples. An additional contributing factor to the discrepancy could be the addition of ‘ballot box stuffing’ preventative measures in the second phase of data collection, which enabled us to prevent individuals from completing our same study multiple times. Therefore, while our two replication studies were otherwise identical in methodology, we believe that as a result of these additional preventative measures, the data yielded from our second sample may be more reliable.

The findings of our replication studies provide some limited support to the idea posited by Côté *et al.* that the tendency of high SES individuals towards utilitarian decision-making is, in part, driven by a lack of empathy^3^. It is thus important to consider the potential theoretical implications of this effect. This finding complicates the depiction of high-class individuals as callous; they are ultimately oriented towards benefitting the greatest number of individuals possible, but this strategy may be rooted in a lack of empathy. The present results provide a promising springboard for future interventions aimed at mitigating the potential ill effects of this type of decision-making. It is possible that the elicitation of empathy could be utilized in order to prevent the alienation and disenfranchisement of individuals who may be adversely affected by utilitarian decisions in real-world contexts.

There are several limitations to our research that are worth discussing. Firstly, participants were recruited via MTurk. While MTurk provides a convenient medium for researchers looking to administer online studies, MTurk samples do have shortcomings. One potential limitation is the evolution of the MTurk population itself; some research has suggested that users’ increased familiarity with psychological studies has diminished the effectiveness of manipulations, a factor which could compromise the replicability of studies^10^. Such familiarity with psychological studies could potentially lead to participants’ suspicion of the study’s hypotheses and inadvertently lead to biased results. This does not, however, fully account for the discrepancy in results between our two samples.

From an analysis standpoint, another limitation of this study was that our primary analyses only allow us to report our replication attempts as successful or unsuccessful. An alternative would be the use of Bayesian methods. If the original data set was acquired, Bayes factors would allow for the investigator to quantify the (relative) evidence for a model (i.e., hypothesis) within the observed data, by not only taking into account the null model, but also the alternative model with which it could be replaced. Therefore, by reporting a Bayes factor there is no need to conclude whether the result showed a successful replication or not. While this was not within the scope of this replication attempt, we feel this would be a valuable future endeavour for other investigators who wish to further examine the replicability of this study.

Another limitation is that our samples do not include individuals who did not have access to MTurk or chose not to use it. While we obtained a normal distribution on the measure of SES, MTurk users of each SES group may not be representative of their counterparts in the general population. These limitations involved with using MTurk samples call for further examination of the relationship between SES and utilitarian judgement and the role that empathy plays in this process, using a different sampling method, in order to increase the generalizability of the findings. Finally, researchers in online studies do not have control over how respondents interpret instructions. Thus, future researchers should look to further test the association between SES, utilitarian judgment, and empathy in both lab and real-world settings. It would be fascinating to investigate the potentially far-reaching societal implications of this area of research.

In conclusion, the present research can speak to the value of replication in the interpretation of research findings. The fact that our first attempt did not successfully replicate the results of Côté *et al.*^3^, but both our second attempt did, and the meta-analysis on the three independent samples (original study, replication attempt #1, and replication attempt #2) suggested the interaction effect was non-zero but potentially very small, points to the importance of conducting more than one replication before reaching any conclusions about the original study. Rejecting a research idea based on a single replication attempt may be equally as unreliable as claiming an effect to be true on the basis of a single non-replicated study.

## Additional Information

**How to cite this article**: Babcock, S. *et al.* Two replications of an investigation on empathy and utilitarian judgement across socioeconomic status. *Sci. Data* 4:160129 doi: 10.1038/sdata.2016.129 (2017).

**Publisher’s note**: Springer Nature remains neutral with regard to jurisdictional claims in published maps and institutional affiliations.

## Supplementary Material



## Figures and Tables

**Table 1:** Data Records.

**Source**	**Subjects**	**Sample size**	**Protocol**	**Experimental Manipulation**	**Data**	**Data Format**
Replication Sample 1	MTurk Participants	*N*=226	Questionnaire	Empathy Induction	https://osf.io/tekuw/	.sav
Replication Sample 2	MTurk Participants	*N=*301	Questionnaire	Empathy Induction	https://osf.io/tekuw/	.sav

**Table 2:** Comparison of Control Variables between the Original and Replication Studies.

**Control Variable**	**Original Study (*****n*****=91)**	**Replication Sample 1 (*****n*****=226)**	**Replication Sample 2 (*****n*****=295)**
Age	Range: 18 to 67	Range:18 to 70	Range: 18 to 78
	*M*=34.65, s.d.=12.3	*M*=35.36, s.d.=11.25	*M*=34.85, s.d.=11.15
Gender	Male: 38%	Male: 57%	Male: 43%
	Female: 60%	Female: 42.5%	Female: 57%
Ethnicity	Caucasian: 71%	Caucasian: 76%	Caucasian: 73%
	Asian American: 4%	Asian American: 9%	Asian American: 12%
	African American: 4%	African American: 8%	African American: 6%
	Latino: 2%	Hispanic: 5%	Hispanic: 5%
	Other: 17%	Other: 1%	Other: 4%
Political Orientation	*M*=3.18, s.d.=1.55	*M*=3.53, s.d.=1.78	*M*=3.51, s.d.=1.61
Religiosity	*M*=3.21, s.d.=2.02	*M*=3.14, s.d.=2.14	*M*=3.36, s.d.=2.06
Household Income	Median=4	Median=4	Median=4
	s.d.=1.98	s.d.=1.80	s.d.=2.02
*Note*: One participant preferred not to disclose gender identity in the original sample, and in replication sample 1.			

**Table 3:** Unstandardized Regression Coefficients for Original and Replication Studies, Model 1.

	**Unstandardized Coefficient (s.e.)**			
	**Original**	**Rep. 1**	**Rep. 2**	**Rep. 1+2**
Income	0.27 (0.12)*	0.09 (0.09)	0.15 (0.07)*	0.12 (0.05)
Condition	−0.10 (34)	−0.47 (0.25)^†^	0.06 (0.20)	−0.17 (0.16)*
Condition*Income	−0.34 (0.17)^†^	−0.02 (0.14)	−0.24 (0.10)*	−0.14 (0.08)^†^
*R*^2^	0.06	0.02	0.02	0.01
^†^*P*<0.1, *P<0.05.				
*Note.* Original=the original study by Côté *et al.*^3^; Rep.1=replication sample one; Rep.2=replication sample two; Rep.1+2=replication sample one and two combined.				

**Table 4:** Unstandardized Regression Coefficients for Original and Replication Studies, Model 2.

	**Unstandardized Coefficient (s.e.)**			
	**Original**	**Rep. 1**	**Rep. 2**	**Rep. 1+2**
Income	0.27 (0.13)**	0.10 (0.09)	0.14 (0.07)*	0.11 (0.05)*
Condition	0.11 (0.36)	−0.51 (0.25)*	0.04 (0.19)	−0.21 (0.15)
Condition*Income	−0.35 (0.17)**	−0.01 (0.14)	−0.23 (0.10)*	−0.12 (0.08)
Gender	−0.67 (0.38)^†^	−0.87 (0.25)***	−0.70 (0.20)***	−0.75 (0.15)***
Age	0.03 (0.01)**	−0.02 (0.01)*	−0.01 (0.01)*	−0.02 (0.01)**
Ethnicity	0.32 (0.46)	0.12 (0.30)	0.44 (0.22)	0.31 (0.18)
Religiosity	−0.04 (0.09)	−0.05 (0.07)	−0.06 (0.05)	−0.04 (0.04)
Conservativeness	−0.01 (0.12)	0.06 (0.08)	−0.06 (0.06)	0.002 (0.050)
*R*^2^	0.17	0.11	0.10	0.09
^†^*P*<0.1, *P≤0.05, **P<0.01, ***P≤0.001.				
*Note.* Original=the original study by Côté *et al.*^3^; Rep.1=replication sample one; Rep.2=replication sample two; Rep.1+2=replication sample one and two combined.				
